# Engineering Properties of Novel Vertical Cutoff Wall Backfills Composed of Alkali-Activated Slag, Polymer-Amended Bentonite and Sand

**DOI:** 10.3390/polym15143059

**Published:** 2023-07-16

**Authors:** Zheyuan Jiang, Xianlei Fu, Jianyong Shi, Chi Che, Yanjun Du

**Affiliations:** 1 Jiangsu Key Laboratory of Urban Underground Engineering and Environmental Safety, Institute of Geotechnical Engineering, Southeast University, Nanjing 210096, China; jiangzheyuan@seu.edu.cn (Z.J.);; 2 Key Laboratory of Ministry of Education for Geomechanics and Embankment Engineering, Hohai University, Nanjing 210024, China

**Keywords:** vertical cutoff wall backfill, polyacrylamide cellulose, hydraulic conductivity, strength

## Abstract

The workability, hydraulic conductivity, and mechanical properties are essential to contaminant containment performance of cementitious backfills in vertical cutoff walls at contaminated sites. This study aims to investigate the engineering properties of a novel vertical cutoff wall backfill composed of reactive magnesia (MgO)-activated ground granulated blast furnace slag (GGBS), sodium-activated calcium bentonite amended with polyacrylamide cellulose (PAC), and clean sand (referred to as MSBS-PAC). Backfills composed of MgO-activated GGBS, sodium-activated calcium bentonite, and clean sand (referred to as MSBS) were also tested for comparison purposes. A series of tests were conducted which included slump test, flexible-wall hydraulic conductivity test, and unconfined compression test. The pore size distributions of two types of backfills were investigated via the nuclear magnetic resonance (NMR) technique. The results showed the moisture content corresponding to the target slump height was higher for MSBS-PAC backfill than that for MSBS backfill. The MSBS-PAC backfill possessed lower pH, dry density, and higher void ratio at different standard curing times as compared to MSBS backfill. The unconfined compressive strength and strain at failure of the MSBS-PAC backfill were noticeable lower than those of the MSBS backfill. In contrast, the hydraulic conductivity of MSBS-PAC backfill was approximately one order of magnitude lower than that of the MSBS backfill, which was less than 10^−9^ m/s after 28-day and 90-day curing. Lower hydraulic conductivity of MSBS-PAC backfill was attributed to the improvement of pore structure and pore fluid environment by PAC amendment.

## 1. Introduction

Vertical cutoff walls with excellent hydraulic conductivity have been extensively used to contain contaminated groundwater at worldwide various contaminated sites and landfill sites (municipal solid waste landfill, hazardous waste landfill, and simple landfill et al.) [1,2,3]. Vertical cutoff walls serve to intercept pathways of pollution and separate sources of contamination from vulnerable receptors [1,4]. Depending on the composition of backfill materials, they are primarily classified as soil-bentonite (SB), soil-cement (SC), cement-bentonite (CB), and soil-cement-bentonite (SCB) [5,6,7]. Compared with SB backfills, which are insufficient to handle external loads due to limited strength, SCB backfills with a specific strength have gained widespread utilization in sites with specific strength requirements [5,8]. Moreover, the utilization of SCB backfills, which involves reusing on-site soil resources, offers a more cost-effective approach compared to CB backfills with similar strength. The construction of CB backfills generates additional processes for the transportation and disposal of excavated soil [1]. However, ordinary Portland cement (OPC) as the primary functional material in SCB and CB backfills results in negative engineering consequences including large carbon dioxide emissions, high energy consumption, and limited chemical compatibility [9,10]. To overcome these challenges, various industrial by-products are used in land remediation and ground improvement projects with extensive application, including but not limited to ground granulated blast furnace slag (GGBS) [10,11,12,13]. It is noted that GGBS has been successfully employed as a partial substitute for cement in backfills with low hydraulic conductivity [14].

Recently, Wu et al. [8,15,16], Fu et al. [17], and Ni [18] developed an MSBS backfill with the merits of lower hydraulic conductivity, superior chemical compatibility, and greater environmental friendliness compared to the conventional backfills consisting of sand and cement, sand and cement-activated slag regardless of bentonite inclusion. The MSBS backfill consisted of reactive MgO, GGBS, conventional bentonite, and sand. Upon hydration, GGBS was activated by MgO and, therefore, multi-valent cations including calcium and aluminum would be released from the MgO-activated GGBS. Additionally, magnesium could be released from the hydrated MgO. The pH of the hydrated MgO-activated GGBS system is alkaline (10.1~10.8) [16]. The presence of multi-valent cations and alkane conditions in the MSBS backfill pore fluid may impose a negative impact on the hydration of the bentonite and mitigate its pore filling and “sand-coating” effects and maintenance of relatively low hydraulic conductivity [8]. The hydraulic conductivity of MSBS backfill specimen with 28-day curing in tap water ranged from 8.5 × 10^−10^ m/s to 4.8 × 10^−9^ m/s [8]. Wu et al. [8] indicated that increasing MgO and GGBS contents would increase the unconfined compressive strength of MSBS backfills while reducing hydraulic conductivity permeated with Pb-Znco-existed solution (concentration of Pb and Zn were 4.83 × 10^−4^ mmol/L and 7.7 × 10^−2^ mmol/L) or sodium sulfate solution (30 mmol/L). Ni [18] found that increasing the contents of MgO and CB would reduce the hydraulic conductivity of MSBS backfills permeated with synthetic landfill leachate. On the other hand, Wu et al. [8,16] and Ni [18] indicated that increasing CB content yielded a decrease in the unconfined compressive strength of MSBS backfills. Moreover, the employment of MSBS backfill resulted in cost savings of approximately 15.33~16.9% and a reduction in CO_2_ emissions of 84.7~85.1% compared to the conventional Ordinary Portlant cement-based backfills [8].

It is noted that the bentonite used in the ordinary MSBS backfill is conventional sodium-activated calcium bentonite (CB), due to the lack of high-quality natural sodium bentonite (NaB) in China and India [19]. The hydraulic conductivity of ordinary SB backfills consisting of sand and CB dramatically increased when exposed to landfill leachate-impacted groundwater and heavy-metal-impacted groundwater [17,20]. Previous studies indicated that superabsorbent hydrogel additives with high water absorption capacity could reduce the hydraulic conductivity and enhance the pollutant containment of bentonite-based engineered barrier materials [21,22,23]. A hydrophilic polymer such as cross-linked superabsorbent polymer (SAP) as an internal curing material was found to be able to reduce the autogenous shrinkage and change the pore size distribution and mechanical properties of cement-based materials [24,25]. Polyanionic cellulose (PAC), a commonly used industrial hydrogel and linear anionic polymer, can increase the viscosity of bentonite suspensions, which is a water-soluble cellulose ether polymer derivative obtained by the chemical modification of natural cellulose [26,27,28]. Moreover, PAC was found to be a promising additive in improving the chemical compatibility of conventional bentonite and backfill consequently [29]. Du et al. [27] and Fu et al. [28] have employed PAC (2% dry weight) as an additive for bentonite to reduce the hydraulic conductivity and improve the chemical compatibility of the bentonite filter cake under heavy metal exposure conditions. Shen [29] showed that PAC addition considerably reduces the hydraulic conductivity of SB backfill to tap water and heavy-metal-impacted groundwater. The reasons are attributed to the pore-filling effect caused by the PAC hydrogel and the “consumption” of multi-valence cations that are aggressive to the thickness of the diffuse double layer (DDL) of montmorillonite mineral in the conventional bentonites [27,28]. Using PAC-amended bentonite to replace conventional bentonite is believed to be effective in tackling this issue. However, to date, no studies have been conducted to investigate if PAC amendment can result in lower hydraulic conductivity of MSBS vertical cutoff wall backfill.

Accordingly, this paper aimed to assess the engineering properties, including workability, hydraulic conductivity, and mechanical properties of PAC-amended and unamended MSBS backfills, based on the results of a series of laboratory experiments including tests of slump, hydraulic conductivity, unconfined compression, and nuclear magnetic resonance (NMR). The results are useful to facilitate the strategy of environmental risk of control of contaminated groundwater using vertical cutoff walls. 

## 2. Materials and Methods

### 2.1. Materials

Sand sampled from the river floodplain of the Yangtze River, Nanjing, Jiangsu Province, China, was employed as the source material of the backfill. The sand was initially dried at a temperature of 105 °C until its mass remained unchanged, followed by sieving through a No. 18 sieve (1 mm). The sand had a coefficient of uniformity (*C*_u_) of 1.78 and a coefficient of curvature (*C*_c_) of 1.00 [20]. The bentonite used was commercially available conventional sodium-activated calcium bentonite (CB) produced in Jianping, Liaoning Province, China, presented as a yellow-brown powder [20]. The particle size of the CB was less than 0.075 mm, with a specific gravity of 2.71, a liquid limit of 335%, a plastic limit of 49%, a plasticity index of 286, a montmorillonite content of 76.2%, and a cation exchange capacity of 61.23 mmol/100 g. According to ASTM D2487 [30], the sand and CB were classified as poorly graded sandy soil (SP) and fat clay (CH), respectively. The ground granulated blast furnace slag (GGBS) was S95-grade GGBS [31], which was produced by Nangang K.Wah Co., Ltd. (Nanjing, Jiangsu Province, China). The GGBS appeared as a gray-white powder with a particle size of less than 0.075 mm, a specific gravity of 2.89, and a specific surface area of 0.428 m^2^/g. The lightweight reactive magnesia (MgO) was supplied by a chemical plant in Shanghai, China, which was a white powder with a MgO content of 78.5%, activity of 90~100 s, density of 3.56 g/cm^3^, and pH of 10.59. The polyanionic cellulose (PAC) was produced by Hunan Pujie Co., Ltd. (Changsha, Hunan Province, China) [28]. PAC had a particle size of less than 0.15 mm, specific gravity of 1.26, weight average molecular weight of 1,730,000, apparent viscosity of 35 mPa·s, a degree of substitution of 1.4, and a pH of 7.3.

### 2.2. Preparation of Backfills

Two backfills were prepared, namely the MSBS and MSBS-PAC backfills. The MSBS backfill consisted of MgO, GGBS, sand, and CB. The MSBS-PAC consisted of MgO, GGBS, sand, and PAC-amended CB. According to previous studies [8,15,16,17,28], the dry weight of CB in the backfill material was 10% of that of sand, while the dry weight of GGBS and MgO was 5% of that of sand, i.e., dry weight ratio of GGBS to MgO was controlled at 9:1. The PAC was selected as an additive with a dosage of 2% (dry weight ratio of PAC to CB). Table 1 shows the mixed proportions of the backfills. Moreover, for adjusting the slump height of the backfill, the CB or PAC-amended CB slurry was used, which consisted of 10% CB or PAC-amended CB and 90% tap water. The pH and EC of tap water were 7.35 and 0.15 mS/cm, respectively.

The preparation procedures for the MSBS-PAC and MSBS backfills were as follows: (1) Appropriate amount of CB with or without PAC were mixed thoroughly to form a dry “Mix A”. (2) Tap water was added into Mix A to obtain PAC-amended or unamended CB slurries. (3) Sand, GGBS, MgO, and CB with or without PAC were mixed thoroughly to form as dry “Mix B”. (4) Mix B was blended with bentonite slurry to obtain fresh backfill and adjust the moisture content of backfill to reach the target slump height. (5) The backfill was layered and filled into a cylindrical rigid mold with a diameter and height of 50 mm and a diameter of 50 mm and a height of 100 mm, respectively. Noted that a backfill layered into a mold with the same diameter and height mold was prepared for the flexible-wall hydraulic conductivity test, while a backfill with double the height to diameter ratio was prepared for the unconfined compression test. The concrete spring vibration machine was adopted to remove the entrapped air in the backfill. (6) The backfill was cured in a standard curing room with a curing temperature of 20 °C and relative humidity of 98%.

### 2.3. Testing Methods

The workability of the backfills was assessed through a slump test as per ASTM C143 [32]. Fresh backfill specimens were tested immediately after preparation, aiming for a slump height between 100 and 200 mm, as recommended by Ryan [1]. In this study, a target slump value of 150 mm was selected for preparing the backfills, which was consistent with Wu et al. [8,16]. After curing for specified curing times, i.e., 0, 7, 14, 28, 60, and 90 days, the specimens were ground and mixed with distilled water at a water-to-solid ratio of 1:1. The resulting mixture was then used to determine the pore water pH, following the method recommended by Wu et al. [8]. A pH meter (HORIBA D-54) was applied to measure pH.

The hydraulic conductivity (*k*) of backfill was determined using tap water as the permeating liquid after 28-day and 90-day curing as per ASTM D5084 [33]. According to Ryan et al. [1] and the Institution of Civil Engineers (ICE) [34], the 28-day and 90-day *k* of SCB backfill are less than 10^−8^ m/s and 10^−9^ m/s, while the Ministry of Industry and Information Technology (MIIT) [35] recommends 10^−9^ m/s as the 28-day *k* for the backfills consisted of cementing constituents including SCB and MSBS. Prior to the flexible-wall hydraulic conductivity test, these specimens were sufficiently saturated by applying a vacuum pressure of 80 kPa for 24 h [8,16]. A constant hydraulic gradient of 150 was maintained, which corresponded to the seepage pressure of 75 kPa. The cell pressure and seepage pressure were set at 162.5 kPa. Both cell pressure and seepage pressure were lower than the yield stress of the specimens to prevent any structural damage. It is noted that volume changes during the tests were found to be negligible, which was because the yield stress of the specimens fell within the range based on the relationship of 1.4~2.2 *q*_u_ [36]. During the permeation process, the environmental temperature was controlled at 22 ± 2 °C. The termination criteria for the hydraulic conductivity test were as follows [33]: (1) the ratio of outflow to inflow fell within the range of 0.75~1.25; (2) the hydraulic conductivity remained steady. Steadiness was determined by observing the hydraulic conductivity vs. time, ensuring that there were no significant upward or downward trends. For *k* values greater than or equal to 10^−10^ m/s, steadiness was achieved if four or more consecutive hydraulic conductivity determinations fell within 25% or better. For *k* values less than or equal to 10^−10^ m/s, steadiness was achieved if four or more consecutive hydraulic conductivity determinations fell within 50% or better [33].

Unconfined compression tests were conducted on the MSBS and MSBS-PAC backfills according to ASTM D4219 [37]. The 28-day *q*_u_ of backfill should exceed the limit value of 100 kPa prescribed by ICE [34] and MIIT [35]. The loading rate of strain was set as 1%/min. The unconfined compression tests were performed on the specimens with 7-day, 14-day, 28-day, 60-day, and 90-day curing. Nuclear Magnetic Resonance (NMR), a non-destructive test method, was extensively used for the analysis of the pore size distribution s of porous media [38]. By detecting the relaxation times of pore fluids, the size of pore structures of porous media can be assessed [39]. The MacroMR12-150V-I large-bore NMR analysis and imaging system (Suzhou Newmai Company, Suzhou, China) was adopted in this study. The MSBS and MSBS-PAC backfill specimens with 90-day curing were subjected to NMR analyses immediately after they were permeated with tap water. The specimens had a diameter and height of 50 mm. All the specimens were not disturbed before NMR analyses.

## 3. Results and Analyses

### 3.1. Workability

**Figure 1:**
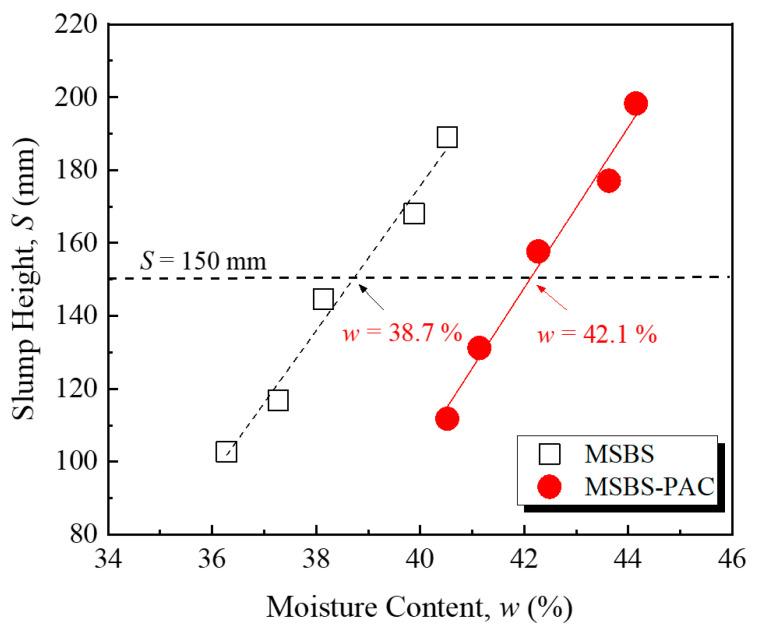
Variation of slump height of backfills with moisture content.

Figure 1 shows the relationship between slump height and moisture content of the backfill material. In this study, the target slump of each backfill was controlled at the median value of 150 mm within the standard slump height range [8,16]. The moisture content of MSBS and MSBS-PAC backfills at the target slump height was 38.7% and 42.1%, respectively. The PAC amendment significantly increased the moisture content of the backfill material corresponding to the target slump height. Compared to the MSBS backfill, the MSBS-PAC backfill showed a 9% increase in moisture content, which was due to the hydrophilic functional groups of PAC polymer including hydroxyl and carboxyl functional groups [11]. A large number of hydrophilic functional groups of PAC promoted backfill water absorption, which thereby increased the moisture content of the backfill corresponding to the target slump height [40].

### 3.2. pH, Void Ratio, and Dry Density

**Figure 2:**
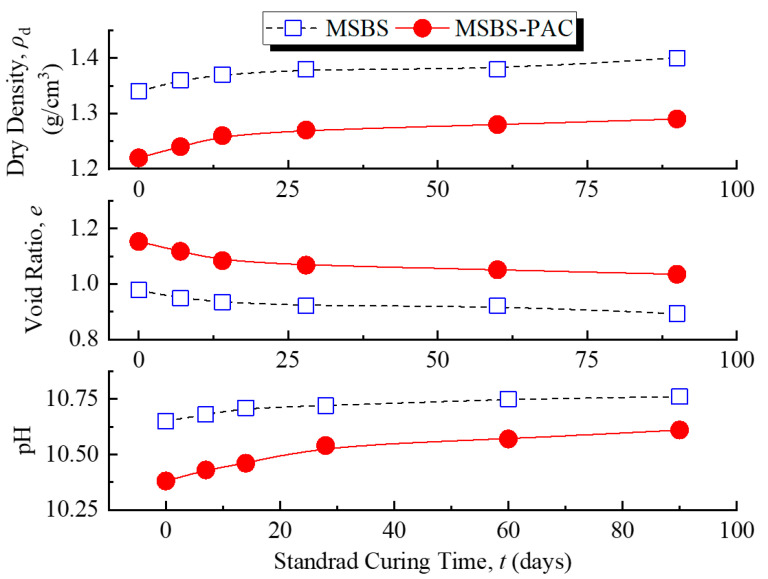
pH, void ratio, and dry density of backfills at different standard curing times.

Figure 2 reveals the changes in pH, void ratio, and dry density of MSBS-PAC and MSBS backfills at different standard curing times. The pH and dry density of the backfills gradually increased with the increase in curing time, while the void ratio gradually decreased. This was consistent with the results from Wu et al. [8,16] and was in line with the change of properties of cementitious materials with curing time. It is noted that the addition of PAC decreased the pH of the backfill material. The void ratio of MSBS-PAC backfill was greater than that of MSBS backfill, while the dry density was lower. Compared to the dry density of MSBS backfill, the dry density of MSBS-PAC backfill was reduced by approximately 8% due to PAC amendment. Furthermore, the void ratio of MSBS-PAC backfill increased by 16%. The observations were attributed to the different initial water contents when two types of backfills were prepared. The initial water contents of backfills were consistent with the moisture contents corresponding to their target slump height.

### 3.3. Hydraulic Conductivity

**Figure 3:**
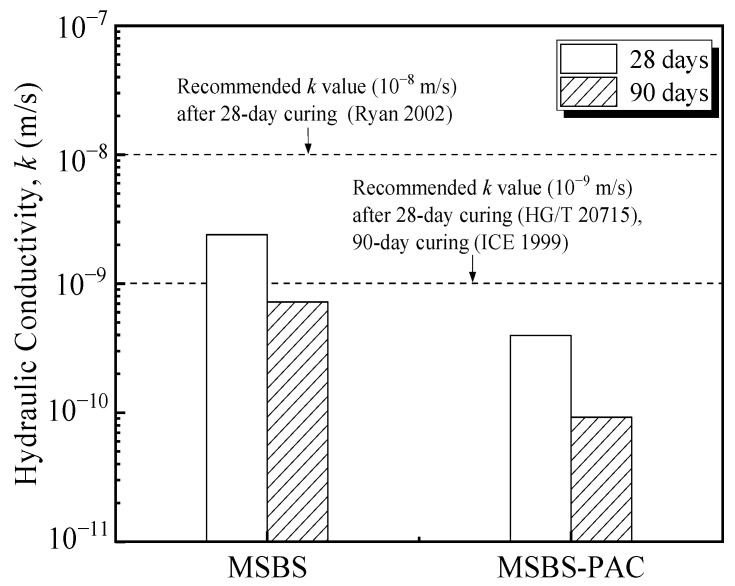
Hydraulic conductivity of backfills permeated with tap water after 28-day and 90-day curing.

The variation of *k* of the backfill materials with curing time is presented in Figure 3. The *k* of both MSBS and MSBS-PAC backfills gradually decreased with increasing curing time, which was consistent with the results from Wu et al. [8,16]. After 90-day curing, the *k* of the backfills decreased by 70% to 77% compared to that after 28-day curing. It is observed that the *k* of MSBS-PAC backfill decreased by approximately one order of magnitude compared to that of MSBS backfill after 28-day and 90-day curing, respectively. The *k* of MSBS backfill was higher than 10^−9^ m/s after 28-day curing, while the *k* of MSBS-PAC backfill was lower than 10^−9^ m/s. The *k* of MSBS backfill material met the values recommended by Ryan et al. [1] (10^−8^ m/s) and the Institution of Civil Engineers (ICE) [34] but did not meet the requirement of the 28-day *k* limit value (10^−9^ m/s) proposed by Ministry of Industry and Information Technology (MIIT) [35]. In contrast, the *k* of MSBS-PAC backfill was less than all recommended values.

### 3.4. Unconfined Compressive Strength, Strain at Failure and E_50_

**Figure 4:**
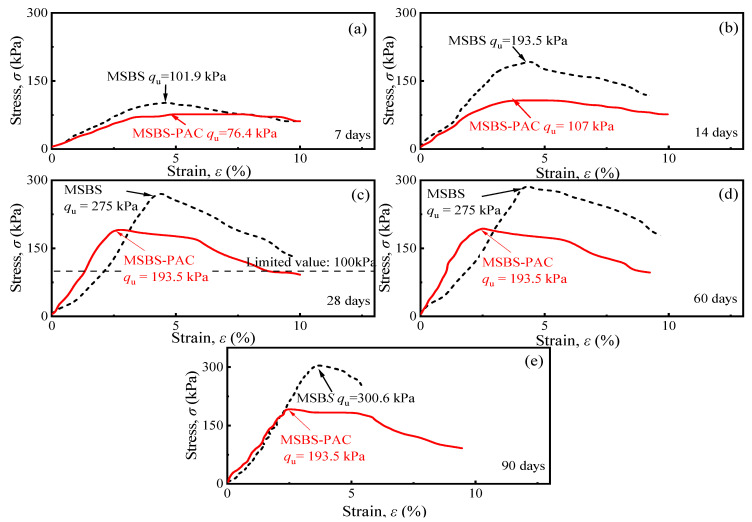
Stress–strain curves of backfills at curing times of (**a**) 7 days; (**b**) 14 days; (**c**) 28 days; (**d**) 60 days; and (**e**) 90 days.

**Figure 5:**
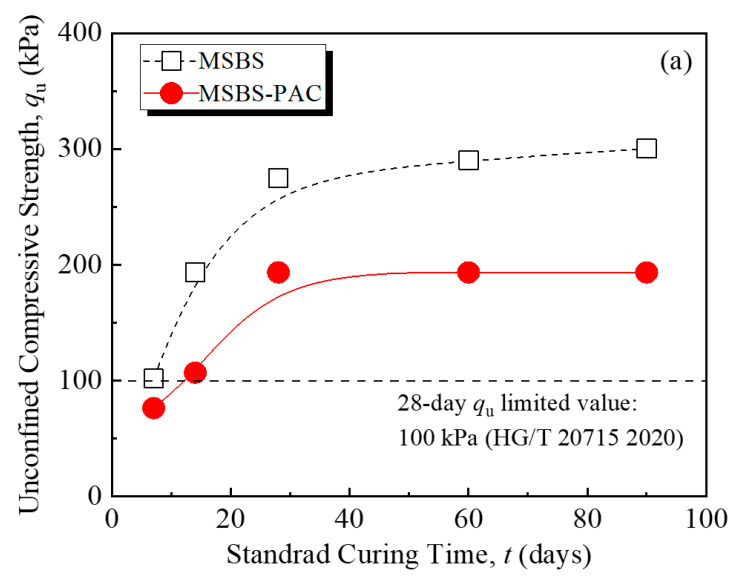

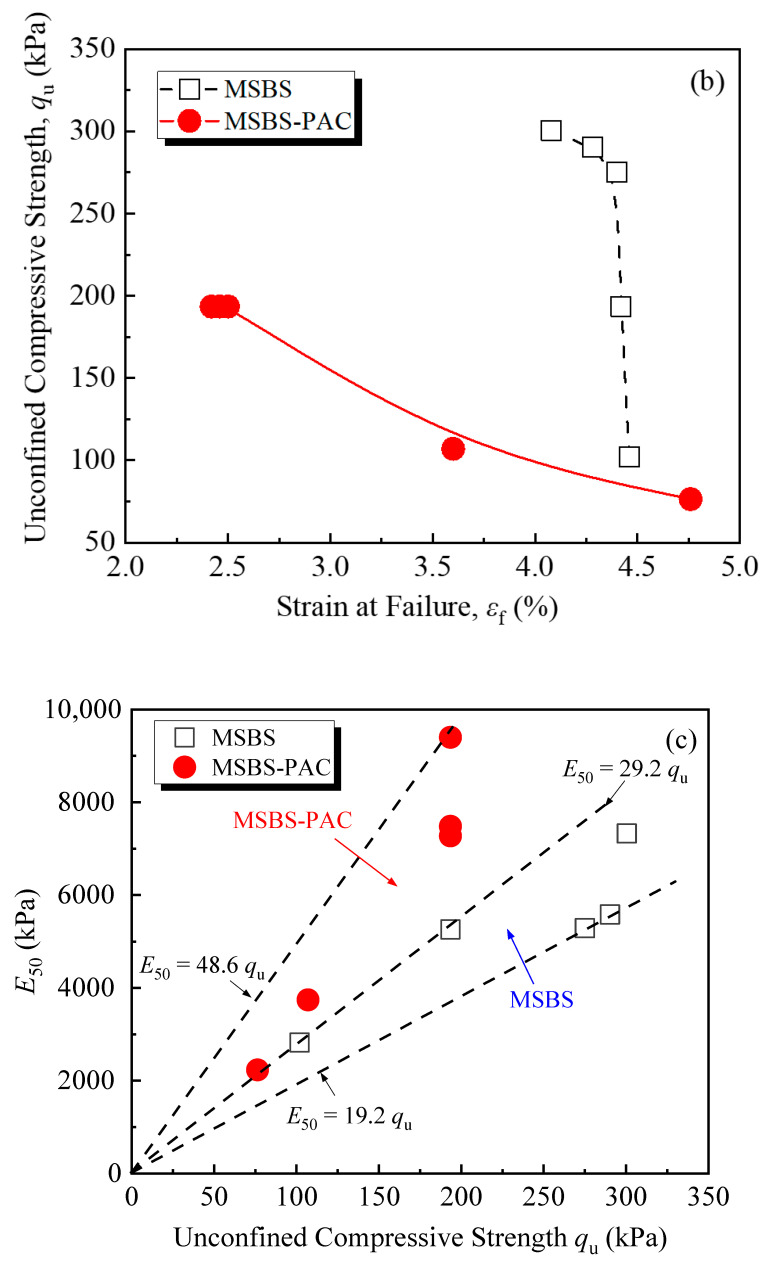
(**a**) *q*_u_; (**b**) *q*_u_ and *ε*_f_; and (**c**) *q*_u_ and *E*_50_ of backfills with different curing times.

Figure 4 shows the stress–strain curves of MSBS and MSBS-PAC backfills at curing times of 7, 14, 28, 60, and 90 days. At each curing time, the peak stress of the MSBS backfill was higher than that of the MSBS-PAC backfill. The unconfined compressive strength (*q*_u_) of the backfills at different curing times is presented in Figure 5a. As the curing time increased, the *q*_u_ of the backfill gradually increased. The *q*_u_ of both backfills at curing 28 days exceeded the limit value of 100 kPa prescribed by ICE [34] and MIIT [35]. The *q*_u_ of MSBS backfill was approximately 33% to 81% higher than that of MSBS-PAC backfill. The *q*_u_ of both backfills gradually stabilized after 28 days of curing. Within 7 to 28 days of curing, the *q*_u_ of the MSBS and MSBS-PAC backfills increased by 170% and 153%, respectively. From 28-day to 90-day curing, the *q*_u_ of MSBS backfill increased by 9%, while the *q*_u_ of PAC-MSBS backfill remained almost unchanged.

Figure 5b presents the relationship between the *q*_u_ and the strain at failure (*ε*_f_) for MSBS and MSBS-PAC backfills. As the *ε*_f_ of the backfill material increased, the *q*_u_ of both backfills decreased. When the *q*_u_ of MSBS backfill was between 102 kPa and 194 kPa, the *ε*_f_ only decreased by 1%. In contrast, when the *q*_u_ of MSBS-PAC backfill increased from 107 kPa to 194 kPa, the *ε*_f_ decreased from 3.6% to 2.4%, a reduction of 33%. When both backfills possessed similar *q*_u_, the *ε*_f_ of MSBS-PAC backfill material was lower than that of the MSBS backfill. *E*_50_ is a deformation modulus that characterizes the ability of materials to resist elastic-plastic deformation, defined as the ratio of stress to strain when stress reaches half of the peak stress [41]. Figure 5c displays the relationship between *E*_50_ and *q*_u_ of different backfills. The *E*_50_/*q*_u_ ratio of MSBS backfill was between 19.2 and 29.2, while that of MSBS-PAC backfill was between 29.2 and 48.6.

### 3.5. Pore Size Distribution

**Figure 6:**
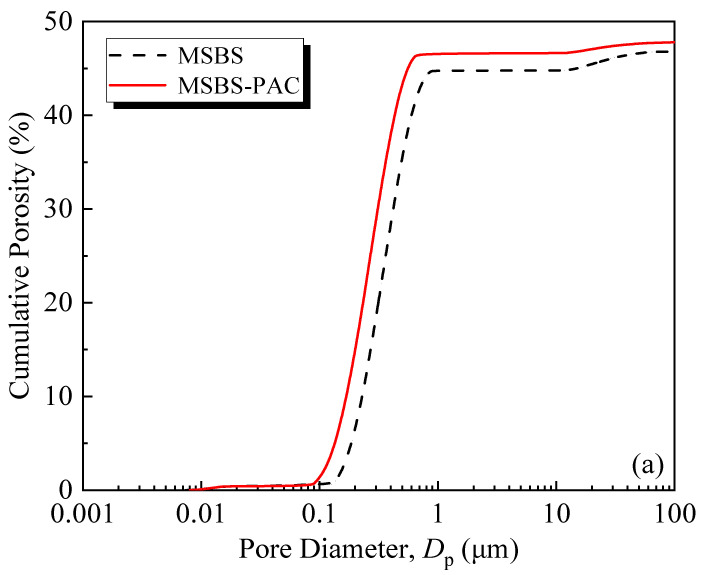

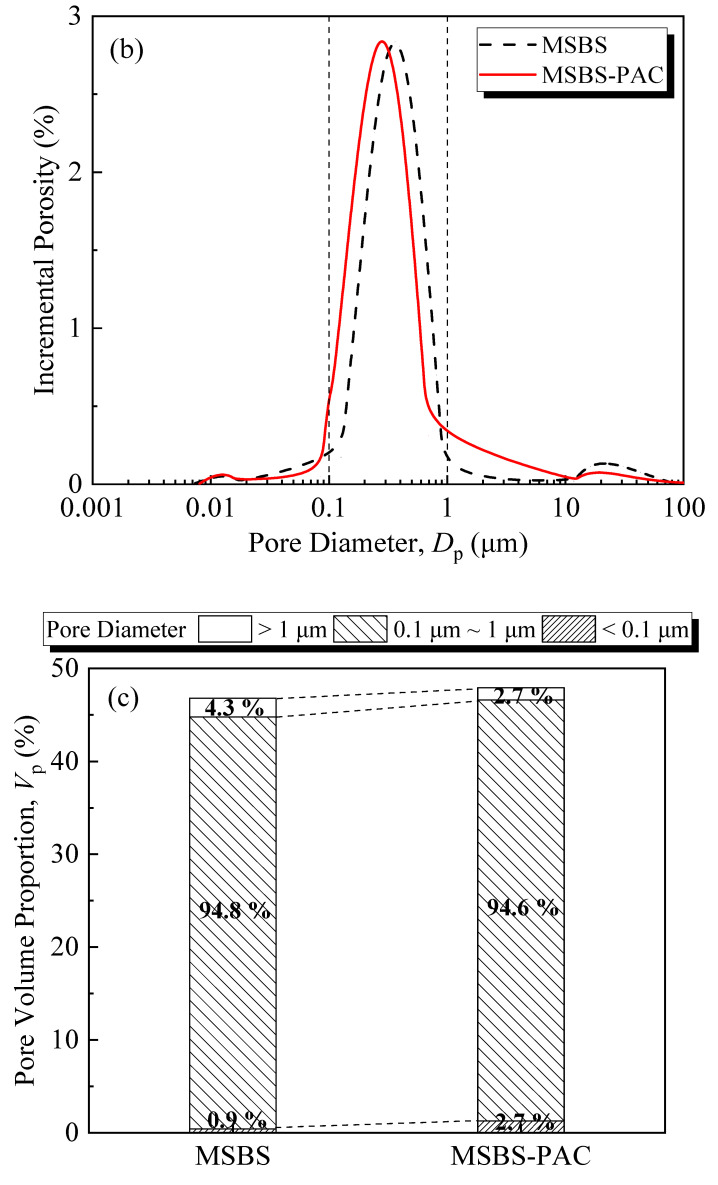
Backfills permeated with tap water: variation of (**a**) cumulative porosity; (**b**) incremental porosity; (**c**) pore volume proportions of different types of pores.

Figure 6 shows the cumulative porosity (Figure 6a), incremental porosity (Figure 6b), and pore volume proportions of different types of pores (Figure 6c) of MSBS and MSBS-PAC backfills with 90-day curing. It is seen from Figure 6a that the PAC amendment increased the cumulative pore volume of MSBS backfill. Compared with the MSBS backfill, the cumulative pore volume of MSBS-PAC backfill material increased by 2.4%. It is noted that PAC amendment increased the initial moisture content at the target slump height, which in turn yielded elevated cumulative pore volume of the backfill. Meanwhile, the peak of the incremental pore size distribution of MSBS-PAC backfill in Figure 6b shifted to the left compared with that of MSBS backfill, indicating a decrease in the pore volume of MSBS-PAC backfill.

The three different pore size ranges of MSBS backfill material were classified as macropores (>1 μm), mesopores (0.1~1 μm), and micropores (<0.1 μm) [16]). As shown in Figure 6c, the volume proportion of pores with pore diameter between 0.1 μm and 1 μm was practically the same for both backfills (94.8% vs. 94.6%), but that in the other two pore size ranges varied. The proportion of macropores for MSBS backfill is higher (4.3% vs. 2.7%), while that of micropores is lower as compared to MSBS-PAC backfill (0.9% vs. 2.7%). After the PAC amendment, the proportion of macropores volume decreased by 37% and that of micropores volume increased by 200%.

## 4. Discussion

### 4.1. Effects of PAC on Hydraulic Conductivity

Based on the *k* values of MSBS and MSBS-PAC backfills cured for 28 and 90 days in Figure 3, PAC amendment reduced the *k* of the backfill by about one order of magnitude. The structure of pores possesses a significant impact on the hydraulic conductivity of porous media [20,42]. According to Figure 2 and Figure 6, the addition of PAC increased the void ratio, micropores volume, and cumulative porosity of the backfills and decreased the macropores volume in the backfill. Compared to mesopores and micropores, the *k* of porous media is mainly influenced by the volume of connected macropores [42,43]. Specifically, with the addition of PAC, the pore size proportion of macropores and micropores for backfill decreased by 37% and increased by 200%, while that of mesopores was practically the same. The observations are attributed to the interactions between PAC, bentonite, and hydration products. The linear anionic polymer PAC molecule possesses a lot of carboxyl (-CH_2_COOH) and hydroxyl (-OH) functional groups [27]. These functional groups on the polymer surface coordinate with the exchangeable cations on the surface of the bentonite and hydration product through water molecules, which are named “water bridges” [23,44]. The hydroxyl functional groups in the PAC lead to the formation of hydrogen bonds in the MSBS-PAC backfill [28], and altered the pore size proportion of macropores and micropores. It is noted that while the increase in the pore volume proportion of micropores reduced the *k* of backfill, elevated cumulative porosity and void ratio could increase the *k* [45]. The phenomenon that MSBS-PAC backfill exhibited lower *k* is attributed to the formation of PAC polymer hydrogel, which possesses a large amount of free water molecules. As the hydrogel of the polymer PAC forms a more narrow and tortuous flow path inside the backfill, it hinders the flow of pore fluid, yielding higher *k* of MSB-PAC backfill as compared to MSBS backfill [21].

It is noted that exposure to high-valence metal ions including aluminum, magnesium, and calcium ions imposes a significant influence on the chemical stability of the montmorillonite in bentonite [14,16]. The high-valence metal ions released from MgO-activated GGBS in the backfill would replace the sodium ions initially adsorbed on bentonite particles, decreasing the DDL thickness of bentonite, and resulting in flocculation of bentonite particles and thus increasing the *k* of the backfill [8,16,20,23]. The addition of PAC to the backfill leads to tight bound of PAC polymer hydrogel to the bentonite particles and therefore forms a protective “thin coating” on the bentonite [28]. The protective “thin coating” could enhance the resistance of backfill against the negative effects of the alkaline condition and multi-valence metal ions. As a result, the *k* of MSBS backfill is reduced after PAC amendment.

### 4.2. Effects of PAC Amendment on Mechanical Properties

Previous studies observed that the addition of hydrophilic polymer (such as cross-linked superabsorbent polymer (SAP)) into cement-based materials adsorbs additional free water molecules and increases the pore volumes, resulting in negative mechanical properties [24,46]. In contrast, Hasholt [47] indicated that the strength of hydrophilic polymer-amended cement-based materials was not changed as compared to unamended materials. Figure 4 and Figure 5 show that the *q*_u_ and *ε*_f_ of the MSBS-PAC backfill were significantly lower than those of the MSBS backfill, while the MSBS-PAC backfill exhibited a higher *E*_50_. The phenomenon is attributed to the following reasons: (a) A large number of hydrophilic functional groups of PAC promote MSBS backfill water absorption [27,28], which is demonstrated by the increased moisture content corresponding to the target slump height as shown in Figure 2, and (b) The higher moisture resulted in a lower dry density, higher void ratio, and pore volume compared to MSBS backfill during the 90-day curing time (Figure 2 and Figure 6a) [46].

## 5. Study Limitations

Further studies are warranted to identify the hydration products of MSBS-PAC backfill and reveal the interactions between PAC and other constituent materials via microscopic test methods including analyses of X-ray diffraction, scanning electron microscopy, and Fourier transform infrared reflection. In addition, the hydraulic conductivity of MSB-PAC backfills permeated with landfill leachate-impacted groundwater and contaminated groundwater should be evaluated.

## 6. Conclusions

The engineering properties (workability, hydraulic conductivity, and mechanical properties) of the MSBS-PAC and MSBS backfills were assessed based on slump tests, flexible-wall hydraulic conductivity tests, and unconfined compression tests. The pore size distributions of MSBS-PAC and MSBS backfills were investigated via nuclear magnetic resonance (NMR) analyses after 90-day curing. Based on the results, the main conclusions could be drawn as follows:

(a) The PAC amendment yielded a 9% increase in moisture content corresponding to the target slump height for the backfill, which was because the carboxyl (-CH_2_COOH) and hydroxyl (-OH) functional groups of PAC combined with free water molecules. The MSBS-PAC backfill possessed lower pH and dry density, and higher void ratio than MSBS backfill.
(b) The PAC amendment reduced the *k* of the backfill by approximately one order of magnitude after 28-day and 90-day curing. With the addition of PAC, the pore volume proportion of macropores in the backfill decreased from 4.3% to 2.7%, while the micropores volume increased from 0.9% to 2.7%. The PAC hydrogel could form a narrow and tortuous flow path inside the backfill, which hindered the flow of permeating liquid and decreased the hydraulic conductivity. In addition, the protective “thin coating” caused by the addition of PAC enhanced resistance against the impact of multi-valence metal ions on bentonite in the backfill.
(c) The *q*_u_ and *ε*_f_ of the MSBS-PAC backfill were lower than those of the MSBS backfill, while the MSBS-PAC backfill yielded a higher *E*_50_. The reasons were attributed to the promotion of water absorption with the hydrophilic functional groups of PAC.

## Figures and Tables

**Table 1:** The mix proportions of backfills (by unit weight of sand, %).

Category ID	Sand	GGBS	MgO	CB	PAC
MSBS	100	4.5	0.5	10	0
MSBS-PAC	100	4.5	0.5	10	0.2

## Data Availability

The data presented in this study are available in this article.
